# Double Trouble: A Case of Gallstone Ileus as a Result of Multiple Obstructive Gallstones

**DOI:** 10.1155/2023/7423380

**Published:** 2023-10-27

**Authors:** Malsha Kularatna, Fransiska Falconer

**Affiliations:** ^1^General Surgical Registrar Bay of Plenty District Health Board, 90 Pakanga Grove, Pyes Pa, Otago, New Zealand; ^2^General Surgical Registrar Tauranga Hospital, 66 Te Horo Drive, Ohope, New Zealand

## Abstract

Gallstone ileus is a rare condition. It accounts for approximately 1% of small bowel obstruction and is more prevalent in the elderly population. It is usually present in relatively comorbid patients posing further operative challenges. The following report investigates the management of two large gallstones resulting in two different points of obstruction. Is double trouble doubly hard to manage?

## 1. Main Text

Mr. X, a New Zealand European 82-year-old male with a background of a previous transient ischaemic attack, hypertension, and osteoarthritis, presented with two months of abdominal bloating, constipation, and generalised abdominal pain. There was no prior history of cholecystitis or known cholelithiasis. He describes intermittent bouts of large-volume vomiting and progressive abdominal distension. He presented two months prior with a similar episode which was deemed to be secondary to an incarcerated umbilical hernia for which he underwent primary repair under general anaesthesia (GA). Of note, the umbilical hernia was found to contain an incarcerated omentum only with no bowel involvement, and the compromised omentum was resected.

**Figure 1:**
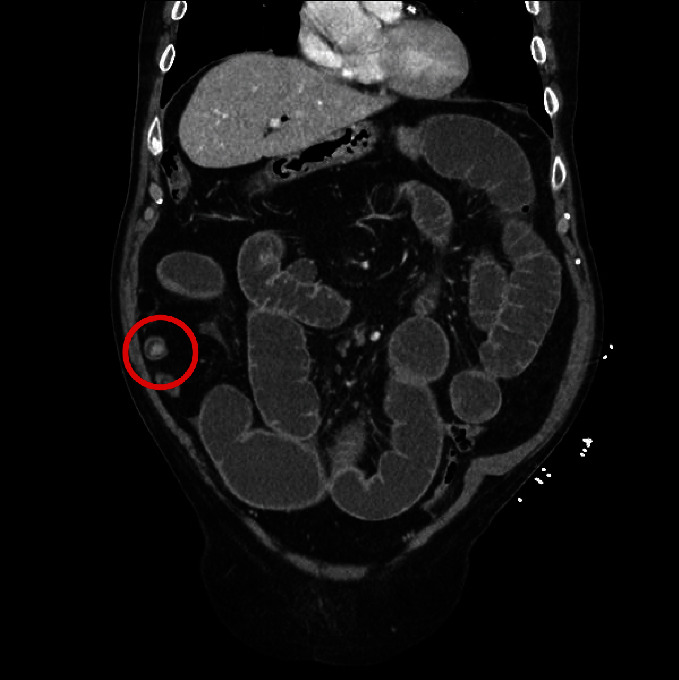
CT imaging demonstrating small bowel obstruction and gallstone impacted in the distal ileum.

On examination, the patient was noted to have a markedly distended abdomen which was generally tender but not peritonitic. His white cell count and c-reactive protein were both slightly raised above normal at 16 and 9, respectively, and his liver functions were normal. Other investigations included a chest X-ray and a CT abdomen-pelvis (Figure 1). A 2 cm gallstone was identified in the distal ileum, causing obstruction and a choledochoduodenal fistula with pneumobilia [1–4]. There was no evidence of perforation.

Mr. X proceeded for laparotomy and enterotomy after the insertion of a nasogastric tube and intravenous line. A midline laparotomy was performed. A large calibre small bowel was identified. A large gallstone was initially encountered in the jejunum on inspection; however, the small bowel appeared to be very dilated distal to this as well. On further inspection, a second stone was also identified (as reported on the imaging) in the distal ileum [5]. The small bowel surrounding these stones was chronically inflamed and thick but appeared viable without any evidence of perforation. Although the distal ileal stone was initially milked proximally with a view of delivering them both out through a single enterotomy, a stricture distal to the jejunal gallstone prevented any further movement. Therefore, two enterotomies were performed approximately 5 cm apart to deliver the two stones which were both approximately 2.5 cm in size. Approximately 2.5 L of turbid fluid was suctioned out of the small bowel following these procedures. Both enterotomies were closed with 3-0 maxon interrupted stitches. The decision was made not to resect the stenosed area of the small bowel as it did appear to be significant. The gallbladder and cholecystoduodenal fistula were not interfered, with during the procedure with the focus being on clearing his mechanical bowel obstruction.

Postoperatively, the patient's diet slowly progressed from sips to free oral fluids as we presumed he would have a prolonged ileus given the chronicity and double stone presentation. However, he was passing flatus by day 3 and progressed well to a normal diet by day 4. He was discharged home on day 7 postadmission and is currently at home awaiting outpatient follow-up to discuss a cholecystectomy; however, given his age, it is unlikely we will go forward with this [6–10].

## 2. Discussion

Gallstone ileus is usually caused by obstruction at the distal ileum and is classically seen in elderly female patients [1, 5, 6, 11]. It is an uncommon cause of mechanical bowel obstruction and is diagnosed with the use of radiological investigation, if not at the time of surgery for the obstruction, where the finding of a gallstone lodged in the bowel confirms the cause [1]. Abdominal imaging that is routinely used includes CT and abdomen X-ray with the findings pertinent for gallstone ileus being pneumobilia, dilated bowel consistent with intestinal obstruction, and presence of gallstones (these signs being known as Rigler's triad) [12].

In this scenario, regarding an elderly gentleman, there was obstruction at both the ileum and the jejunum. Whether his initial presentation of incarcerated umbilical hernia was also perpetuated by the presence of these stones is uncertain, as there was no imaging performed at this point [3].

In this patient's case, the mechanism of gallstone ileus appears to be originally caused by a bout of cholecystitis, as expected as the root cause of all gallstone ileus cases. However, it appears that the subsequent pericholecystic inflammation and resultant adhesions around the biliary systems and bowel that were in close proximity have led to two stones being passed through these adhesions [2, 13].

It would be feasible to assume the second stone was held up at the jejunum, resulting in thickening and stenosis secondary to the hold-up created by the first stone obstructing the distal ileum.

While this presentation was unusual, we can suffice to say the patient made a remarkable recovery given his presentation and having had two GAs within close proximity.
